# Dr. Bigelow

**Published:** 1853-10

**Authors:** 


### DR. BIGELOW.

It is with sincere regret we have heard of the decease of this estimable professionable brother. It is more than twenty years since we first met with him in Vicksburg, Miss. For several years, the Doctor spent his winters in the South, and obtained an enviable reputation for integrity and professionl skill. But for the last few years, he has been principally located at Cleveland in this State, and perhaps part of the time in Newark. Last spring he passed through our city on his way down the river, intending, as we believe, to return in a few weeks, but he lingered too long in the inviting climate of the South, and fell a victim to the scourge which has so desolated that portion of our country. We know not just the time of his decease, or at what point the event occurred. We hope some friend will give us the particulars, or prepare a befitting obituary.

